# The proinflammatory cytokines IL-1β and TNF-α induce the expression of Synoviolin, an E3 ubiquitin ligase, in mouse synovial fibroblasts via the Erk1/2-ETS1 pathway

**DOI:** 10.1186/ar2081

**Published:** 2006-11-14

**Authors:** Beixue Gao, Karen Calhoun, Deyu Fang

**Affiliations:** 1 Department of Otolaryngology-Head and Neck Surgery, University of Missouri School of Medicine, One Hospital Drive, Columbia, MO 65212, USA; 2 Department of Molecular Microbiology and Immunology, University of Missouri School of Medicine, One Hospital Drive, Columbia, MO 65212, USA

## Abstract

The overgrowth of synovial tissues is critical in the pathogenesis of rheumatoid arthritis (RA). The expression of Synoviolin (SYN), an E3 ubiquitin ligase, is upregulated in arthritic synovial fibroblasts and is involved in the overgrowth of synovial cells during RA. However, the molecular mechanisms involved in the elevated SYN expression are not known. Here, we found that SYN expression is elevated in the synovial fibroblasts from mice with collagen-induced arthritis (CIA). The proinflammatory cytokines interleukin (IL)-1β and tumor necrosis factor-α (TNF-α) induce SYN expression in mouse synovial fibroblasts. Cultivation of mouse synovial fibroblasts with IL-1β activates mitogen-activated protein kinases, including extra-cellular signal-regulated kinase (Erk), JNK (c-Jun N-terminal kinase), and p38, while only Erk-specific inhibitor blocks IL-1β-induced SYN expression. Expression of transcription factor ETS1 further enhances IL-1β-induced SYN expression. The dominant negative ETS1 mutant lacking the transcription activation domain inhibits SYN expression in a dose-dependent manner. The activation of both Erk1/2 and ETS1 is increased in the CIA synovial fibroblasts. Inhibition of Erk activation reduces ETS1 phosphorylation and SYN expression. Our data indicate that the proinflammatory cytokines IL-1β and TNF-α induce the overgrowth of synovial cells by upregulating SYN expression via the Erk1/-ETS1 pathway. These molecules or pathways could therefore be potential targets for the treatment of RA.

## Introduction

Rheumatoid arthritis (RA) is a chronic debilitating disease of the joints characterized by leukocyte infiltration, hyperproliferation of synovial cells, and bone destruction. Hyperproliferative synovial fibroblasts play a critical role in the pathogenesis of RA by the following mechanisms: They directly invade bone and cartilage, produce proinflammatory cytokines such as tumor necrosis factor-α (TNF-α) and interleukin (IL)-1β [1], destroy cartilage through the production of metalloproteinase [2], and produce the receptor of nuclear factor-kappa B (NF-κB) ligand, which augments osteoclast activity for bone destruction [3-5]. Therefore, inhibition of the proliferative and/or invasive capacities of synovial fibroblasts should have protective effects against joint destruction.

Synoviolin (SYN), which is also called Hrd1 (3-hydroxy-3-methylglutaryl reductase degradation), was identified by Hampton and co-workers [6] as an E3 ubiquitin ligase in yeast. SYN is a multispanning membrane protein with its C-terminal RING (really interesting new gene) finger domain located in the cytoplasm [6,7]. It has been reported that human SYN is involved in the elimination of two endoplasmic reticulum (ER)-associated degradation substrates, T-cell receptor-α and CD3-δ, via its E3 ubiquitin ligase activity [8]. Ubiquitination is a process that covalently conjugates ubiquitin to the target protein for degradation. This process requires a cascade of three enzymes, E1, E2, and E3. SYN predominantly uses the ubiquitin-conjugation enzyme 7p (Ubc7p) as an E2 but also cooperates with Ubc6p and Ubc1p in ER-associated degradation [9]. SYN is also required for the mouse embryo development because the gene knockout mice die *in utero *at approximately embryonic day 13.5 [10].

By means of immunoscreening with an anti-rheumatoid synovial cell antibody, SYN was identified and cloned as a rheumatoid regulator. Expression of SYN is highly associated with the development of RA. Mice with overexpressed SYN (SYN transgenic mice) develop spontaneous arthropathy. On the other hand, mice with reduced SYN (SYN^+/- ^mice) are resistant to collagen-induced arthritis (CIA). Further *in vitro *study revealed that, through its anti-apoptotic activities, SYN triggers the outgrowth of synovial fibroblasts [11,12]. Therefore, inhibition of the expression of SYN has potential therapeutic benefit in the prevention or treatment of RA. However, the molecular mechanisms involved in overexpression of SYN during RA remain unknown.

In this study, we found that the proinflammatory cytokines, particularly IL-1β, upregulate SYN expression at the transcriptional level. The extra-cellular signal-regulated kinase (Erk)-ETS1 signal pathway is involved in IL-1β-induced SYN expression.

## Materials and methods

### Reagents, antibodies, and plasmids

All the mitogen-activated protein kinase (MAPK) inhibitors used in this study, including the Erk activation inhibitor, PD98059 [13], the c-Jun N-terminal kinase (JNK) inhibitor, SP600125 [14], p38 inhibitor, SB202190 [15], and the NF-κB inhibitor, SN50 [16], were purchased from Calbiochem (San Diego, CA, USA). Antibodies against JNK1, Erk, and p38 were purchased from Promega Corporation (Madison, WI, USA). Murine IL-1β, IL-6, and TNF-α were obtained from BD Pharmingen (San Diego, CA, USA). Anti-actin monoclonal antibody was purchased from Santa Cruz Biotechnology, Inc. (Santa Cruz, CA, USA), and anti-ETS1 was obtained from EMD Biosciences, Inc. (San Diego, CA, USA). Antibodies against Erk, c-Jun, and ATF2, as well as their phosphorylated forms, were purchased from Promega Corporation. Anti-SYN polyclonal antibody was generated in our laboratory by immunization of mice with glutathione S-transferase (GST)-fusion protein of the C-terminal 152 amino acids of SYN. ETS1 full-length cDNA was purchased from American Type Culture Collection (ATCC) (Manassas, VA, USA) (ATCC no. 5844553). The C-terminal DNA binding domain of ETS1 was amplified by polymerase chain reaction (PCR) and subcloned into pEF4 his expression vectors digested by KpnI and Not1.

### Mice and CIA

DBA1 mice (The Jackson Laboratory, Bar Harbor, ME, USA) were bred and maintained in accordance with the guidelines of the institutional animal care and use committee (IACUC), and all the experimental procedures were approved by the IACUC of the University of Missouri (Columbia, MO, USA).

Native bovine collagen II (Worthington Biochemical Corporation, Lakewood, NJ, USA) was emulsified with an equal volume of complete Freund's Adjuvant (CFA). Disease was induced by intradermal injection of DBA1 mice with 50 μl of emulsion containing 100 μg of collagen in CFA. On day 21, the mice were boosted by intradermal injection with 100 μg of collagen in incomplete Freund's Adjuvant. Clinical arthritis was assessed by the following scoring system: grade 0, no swelling; grade 1, mild but definite redness and swelling of the ankle or wrist or digits; grade 2, moderate redness and swelling of ankle and wrist; grade 3, severe redness and swelling of entire paw, including digits; and grade 4, maximally inflamed limb with involvement of multiple joints. Each limb was graded, giving a maximum possible score of 16 per mouse. Approximately 80% of DBA1 mice developed arthritis 40 days after the first injection with collagen (supplemental Figure 1a in Additional file 1), and most of these mice developed severe arthritis with an average score of 12 (supplemental Figure 1b in Additional file 1).

### Isolation of synovial fibroblasts

Synovial tissues were obtained from DBA1 mice as described previously [17]. These joint tissues were minced and incubated with 1 mg/ml of collagenase (Worthington Biochemical Corporation) in serum-free Dulbecco's modified Eagle's medium (DMEM) for 3 hours at 37°C, filtered through a nylon mesh, extensively washed, and cultured in DMEM supplemented with 10% fetal calf serum (Fisher Scientific Co., Pittsburgh, PA, USA), 100 U penicillin, 100 μg/ml streptomycin, and 50 mg/ml L-glutamine in a humidified atmosphere containing 5% CO_2_. After overnight culture, we removed the nonadherent cells, trypsinized the adherent cells split at a ratio of 1:3, and cultured them in medium. Synoviocytes were used from passages 3 to 9 in these experiments, during which they consisted of a homogeneous population of synovial fibroblasts monitored by flow cytometry with less than 1% of CD11b, phagocytic, and Fc receptor II-positive cells.

### SDS-PAGE and Western blotting

We analyzed the expression of SYN by SDS-PAGE and Western blotting as described previously [18]. Synovial fibroblasts were collected from culture dishes and lysed with Nonidet P-40 (NP-40) lysis buffer (20 mM Tris-HCl with pH 7.5, 150 mM NaCl, 1% NP-40, and protease inhibitor cocktail was added freshly) and boiled in 20 μl of Laemmli's buffer (50 mM Tris-HCl, pH 6.8, 30% glycerol, 4% SDS, and 1% β-mercaptoethanol). Samples were subjected to 8% or 10% analysis by SDS-PAGE and electrotransferred onto polyvinylidene difluoride membranes (Millipore, Billerica, MA, USA). Membranes were probed with the indicated primary antibodies (usually 1 μg/ml), followed by horseradishperoxidase-conjugated secondary antibodies. Membranes were thenwashed and visualized with an enhanced chemiluminescence detectionsystem (Amersham Pharmacia Biotech, now part of GE Healthcare, Little Chalfont, Buckinghamshire, UK). When necessary, membranes were stripped by incubation in stripping buffer (62.5 mM Tris-HCl, pH 5.7,100 mM 2-mercaptoethanol, and 2% SDS) for 30 minutes at 70°C with constant agitation, washed, and then reprobed with other antibodies asindicated.

### Real-time reverse transcription-PCR

Total cellular RNA extraction was performed using the RNA purification kit from Promega Corporation. The oligonucleotide primers used for mouse SYN were forward 5-aggcccatgtacctggccatgagg-3 and reverse 5-caggagcgcaggcagctcgtgtg-3. The QuantiTect SYBR Green PCR kit (Qiagen Inc., Valencia, CA, USA) was used. Reactions were loaded on 96-well thin-wall plates and sealed with optical-quality sealing tape. Each reaction was run on an iCycler iQ Multi-Color Real-Time PCR detection system (Bio-Rad, Hercules, CA, USA) under the following conditions: 95°C for 15 minutes (94°C for 30 seconds, 55°C for 30 seconds, and 72°C for 30 seconds) for 40 cycles and 72°C for 3 minutes. Samples were run in triplicate, and relative copy numbers were determined.

## Results

### Elevated expression of SYN during CIA in DBA1 mice

**Figure 1:**
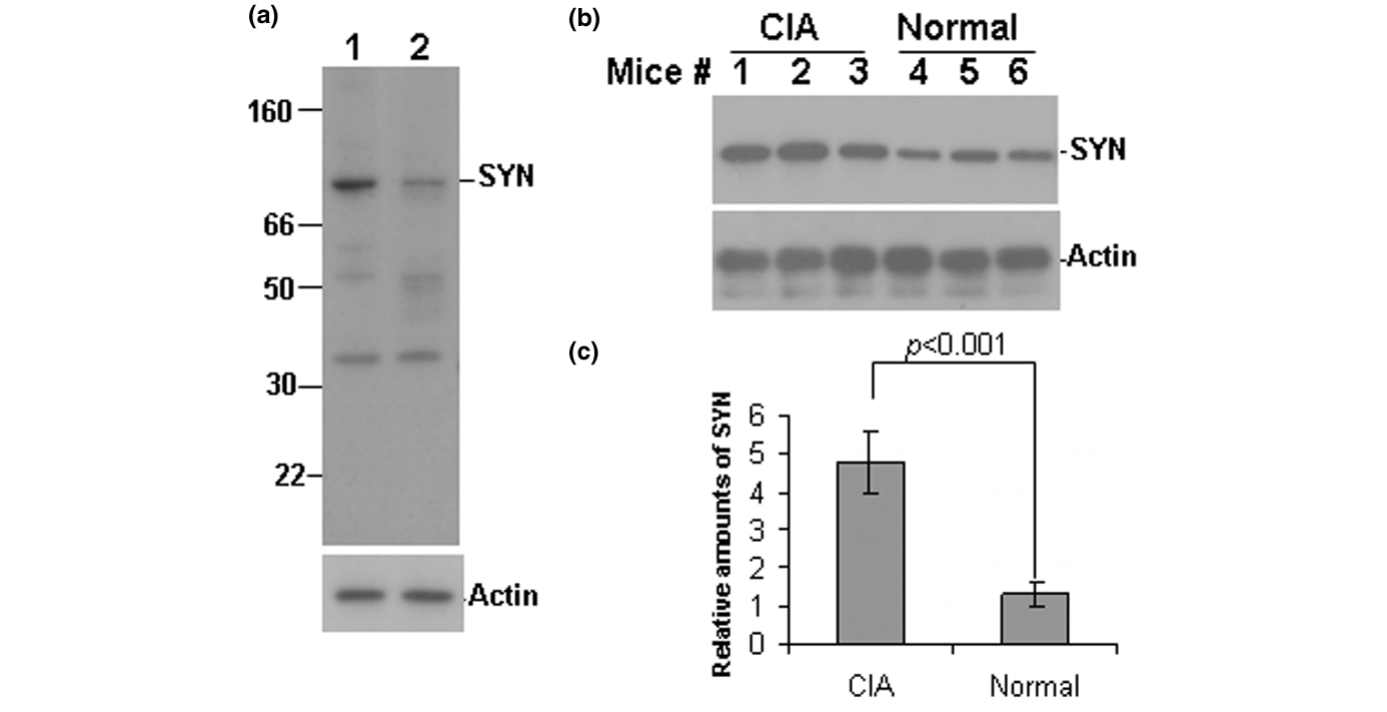
Increased Synoviolin (SYN) expression in the synovial fibroblasts from collagen-induced arthritis (CIA) mice. **(a) **Test of the specificity of anti-SYN antibody. HEK 293 cells were transfected with (lane 1) or without (lane 2) SYN expression plasmids. Lysates from the transfected cells were analyzed by SDS-PAGE and Western blotting using sera from the mice immunized with GST (glutathione S-transferase)-SYN fusion protein (top panel). The same membrane was reprobed with anti-actin (bottom panel). **(b) **Analysis of the protein expression of SYN in mouse synovial fibroblasts. Mouse synovial fibroblasts were isolated from each of three normal or CIA mice. Cells were lysed and subjected to SDS-PAGE and Western blotting using anti-SYN polyclonal antibodies (top panel). The same membrane was reprobed with anti-actin antibody (bottom panel). **(c) **Quantification of SYN expression. The densities of each band in **(b) **were analyzed, and the ratio of SYN/(10^-1 ^actin) was used. Error bars represent three different CIA or normal mice. Statistic analysis indicates that the expression of SYN is significantly increased (*p *< 0.001).

Recent studies demonstrated that SYN functions as a rheumatoid regulator for the overgrowth of synovial tissues in patients with RA [11]. To investigate the molecular mechanisms involved in regulating SYN expression during arthritis development, we compared SYN expression in the synovial fibroblasts from CIA mice with that from normal mice. Because the antibodies against SYN are not commercially available, we first generated anti-SYN polyclonal antibody by immunizing a fusion protein of GST with the C-terminus of SYN. We detected both overexpressed and endogenous SYN in HEK 293 cells by using the sera from GST-SYN-immunized mice (Figure 1a). We therefore used these polyclonal anti-SYN antibodies in this study.

With Western blotting analysis, we found a significantly higher SYN expression in the synovial fibroblasts from CIA mice than that from normal controls (Figure 1b). The mean relative density values (10 × SYN/actin) were 4.8 ± 0.9 for CIA synovial fibroblasts and 1.1 ± 0.3 for control synovial cells (*p *< 0.001) (Figure 1c). These results indicate that SYN expression is upregulated during CIA.

**Figure 2:**
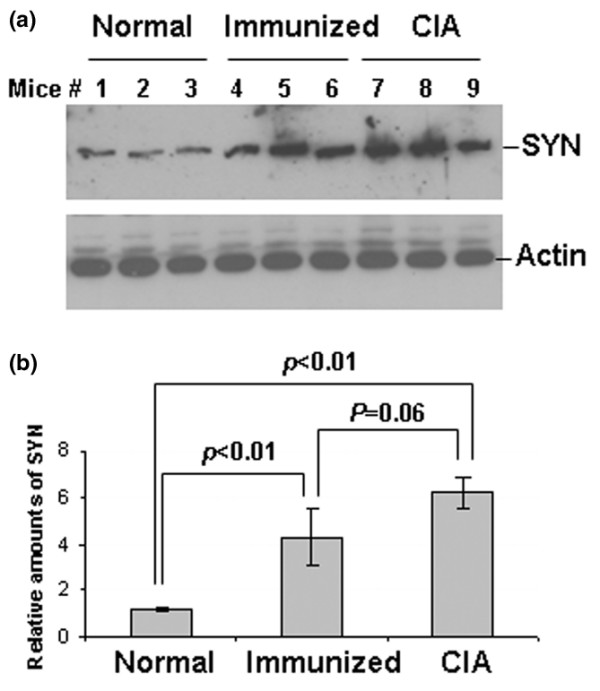
The expression of Synoviolin (SYN) during collagen-induced arthritis (CIA) development. **(a) **Synovial fibroblasts were isolated from normal DBA mice (Normal) and DBA mice 20 days after the first collagen immunization (Immunized) or 10 days after the second immunization when CIA is developed (CIA). The expression of SYN in those synovial fibroblasts was analyzed by Western blotting. **(b) **The densities of each band in **(a) **were analyzed, and the ratio of SYN/(10^-1 ^actin) was used. Error bars represent three different CIA, immunized, or normal mice. Statistic analysis indicates that the expression of SYN is significantly increased during CIA development.

Next, we kinetically analyzed the expression of SYN during the development of CIA. DBA synovial fibroblasts from normal DBA mice, DBA mice 20 days after the first immunization with collagen but without any clinical sign of CIA, and DBA mice 10 days after boosted immunization when these mice developed severe arthritis (score 8 to 12). Surprisingly, the expression of SYN has been significantly upregulated when the DBA mice received only the first immunization and did not develop obvious arthritis. Although statistically not significant (*p *= 0.06), boosted immunization further enhanced SYN expression (Figure 2). These results suggest that upregulation of SYN expression is involved in the development of CIA in mice.

### Induction of SYN expression by IL-1β and TNF-α in mouse synovial fibroblasts

**Figure 3:**
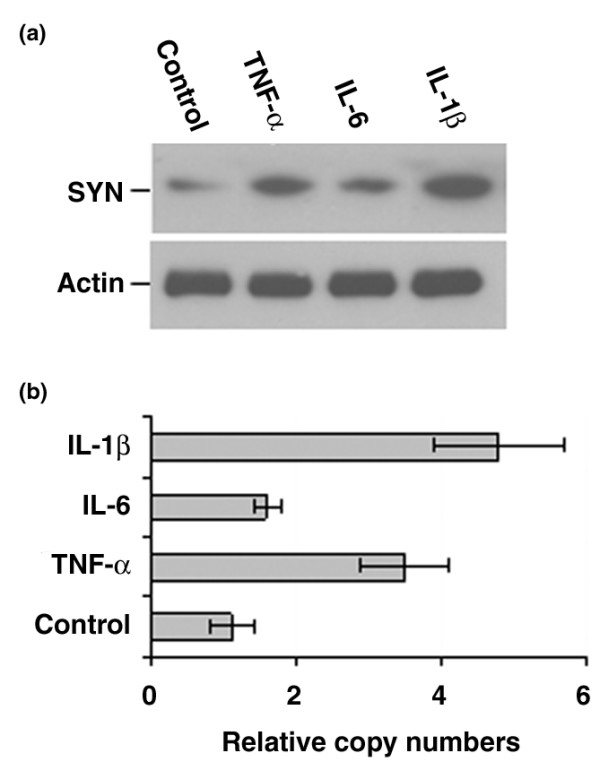
Induction of Synoviolin (SYN) expression by proinflammatory cytokines. **(a) **Mouse synovial fibroblasts were starved for 24 hours and then cultivated for 48 hours with each proinflammatory cytokine, including 10 ng/ml of tumor necrosis factor-α (TNF-α), interleukin (IL)-1β, or IL-6. The expression of SYN was tested by Western blotting by using anti-SYN antibody (top panel). As a control, the protein level of actin was detected using the same membrane (bottom panel). **(b) **The total RNA from the cultured synovial fibroblasts was isolated and reverse-transcribed into cDNA. The levels of SYN cDNA were analyzed by real-time polymerase chain reaction by using SYN-specific primers. Error bars represent the results from three independent experiments (mean ± standard deviation).

The production of proinflammatory cytokines by synovial macrophages and fibroblasts is one reason for synovial cell hyperproliferation. We therefore hypothesized that some of these cytokines may also be responsible for the induction of SYN expression. To test this hypothesis, we first examined the effects of proinflammatory cytokines on the expression of SYN in mouse synovial fibroblasts. Each of 5 × 10^5 ^cells was plated and cultivated in the presence of IL-6, TNF-α, or IL-1β. Forty-eight hours after the addition of each cytokine, the protein levels of SYN were analyzed. As shown in Figure 3a, TNF-α slightly induced the protein expression of SYN with a mean density of 3.1 ± 0.6 compared with 1.1 ± 0.2 of controls from three independent experiments (*p *< 0.05 to control), whereas IL-6 did not affect SYN expression, the mean density of which was 1.3 ± 0.3 (*p *= 0.28). The cultivation of synovial fibroblasts with IL-1β significantly upregulated SYN expression, the mean density of which was 6.2 ± 1.1 (*p *< 0.001). These results indicate that both IL-1β and TNF-α induce SYN expression in mouse synovial fibroblasts.

To determine whether these cytokines induce SYN expression at the transcriptional level, total RNA was isolated from these synovial fibroblasts, reverse-transcribed into cDNA, and examined by reverse transcription (RT)-PCR using SYN-specific primers. Real-time RT-PCR revealed that IL-1β induced SYN expression at the transcriptional level, because the mRNA level of SYN was upregulated by IL-1β. TNF-α had a weaker effect on SYN transcription, whereas IL-6 had no effect (Figure 3b). These results indicate that IL-1β induces SYN expression at the mRNA level in mouse synovial fibroblasts.

### IL-1β and TNF-α induce SYN expression through activation of Erk

**Figure 4:**
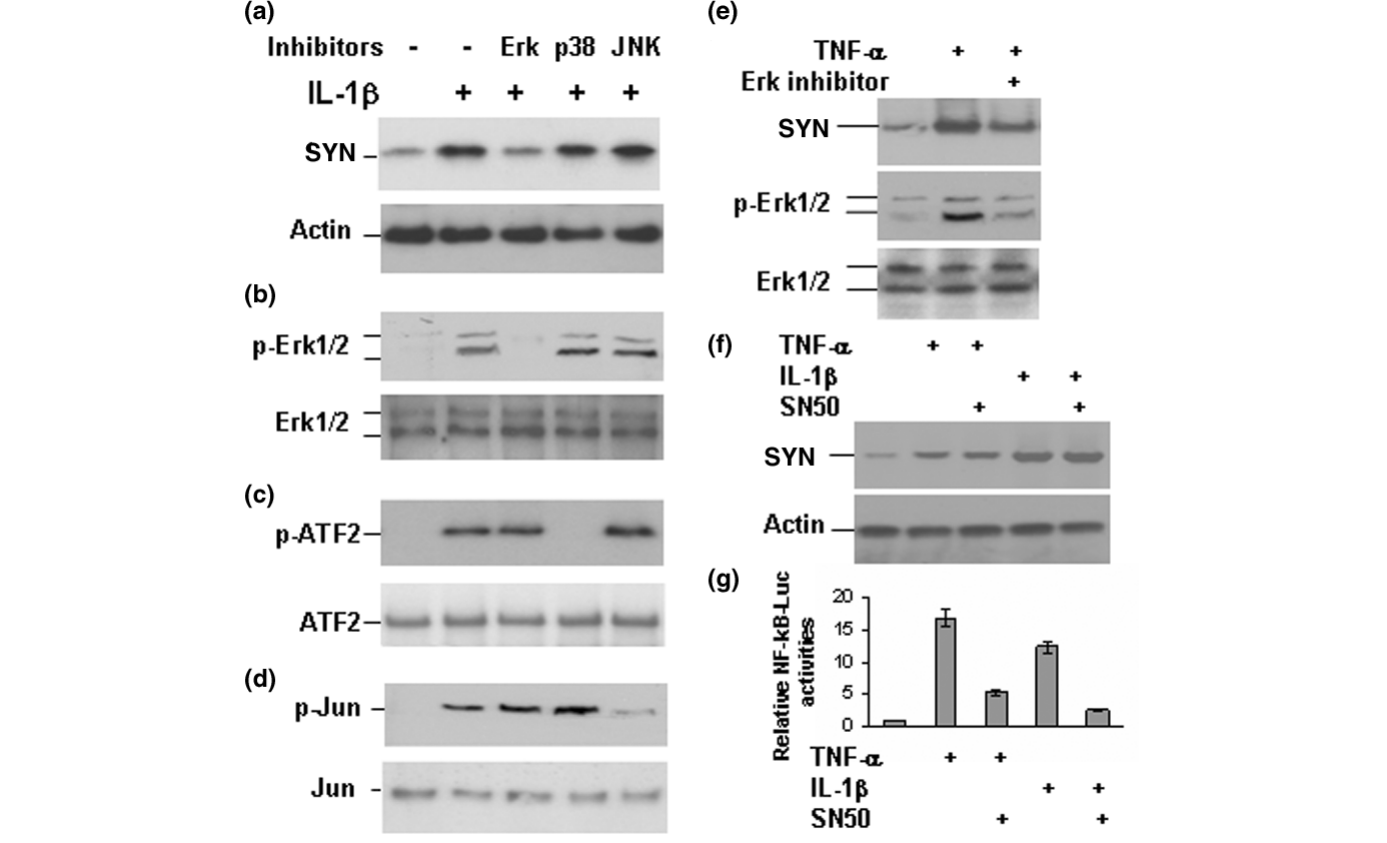
Interleukin (IL)-1β and tumor necrosis factor-α (TNF-α) induce Synoviolin (SYN) expression via the Erk pathway. **(a) **Synovial fibroblasts were partially starved for 24 hours by cultivation of these cells with media that contain 0.5% fetal bovine serum and then cultured with 10 ng/ml of IL-1β. Cells were also treated with each of the MAPK inhibitors. The concentrations of each inhibitor used were as follows: Erk inhibitor PD98059, 20 μM; JNK inhibitor SP600125, 10 μM; p38 inhibitor SB202190, 10 μM; and NF-κB inhibitor SN50, 20 μM. The expression of SYN was examined by Western blotting (top panel). The protein level of actin was reprobed as a control (bottom panel). **(b) **The activation of Erk was analyzed by anti-p-Erk antibody (top panel). The same membrane was reprobed by anti-Erk antibody (bottom panel). **(c) **p38 activation was analyzed by anti-p-ATF2 antibody (top panel). The same membrane was reprobed by anti-ATF2 antibody (bottom panel). **(d) **JNK1 activation was analyzed with anti-phospho-Jun antibody (top panels). The total protein levels of c-Jun were examined using anti-Jun (bottom panels). **(e) **Mouse synovial fibroblasts were starved for 24 hours and then cultured with 10 ng/ml of TNF-α without or with Erk inhibitor. The expression of SYN was analyzed by Western blotting (top panel). The activation of Erk was determined by anti-phosphorylated Erk antibody (middle panel). The protein level of Erk was analyzed by anti-Erk antibody (bottom panel). **(f) **The effect of NF-κB inhibitor on SYN expression. Mouse synovial fibroblasts were transfected with NF-κB-luc reporter, which contains firefly luciferase gene under control of NF-κB. The control plasmid pRL-TK encoding renillar luciferase was also included to correct transfection efficiency. Transfected cells were starved and then cultivated in the presence of 10 ng/ml of IL-1β or TNF-α without or with SN50 for 24 hours. The expression of SYN in these cells was analyzed by Western blotting (top panel), and the same membrane was reprobed with anti-actin (middle panel). **(g) **Parallel prepared cell lysates from **(f) **were used for testing the NF-κB-driven luciferase activity (bottom panel). Error bars represent three different experiments (mean ± standard deviation). Erk, extracellular signal-regulated kinase; JNK, c-Jun N-terminal kinase; MAPK, mitogen-activated protein kinase; NF-κB, nuclear factor-kappa B.

IL-1β induces the growth of synovial fibroblasts by activating the MAPK pathways [19-24]. To elucidate the molecular mechanisms of IL-1β-induced SYN expression, we first tested the activation of all three MAPK members, including Erk, JNK, and p38. Because culture of synovial fibroblasts with 10% fetal bovine serum (FBS) highly activates all these MAPKs, we first starved the mouse synovial fibroblasts for 24 hours with media that contain 0.5% FBS, then IL-1β was added. Erk activation was analyzed with anti-phosphorylated Erk. The phosphorylations of c-Jun and ATF2 were used as reporters for the activation of JNK1 and p38, respectively [19]. The activation of these MAPKs was not detectable after starvation (Figure 4b–d, lane 1). Cultivation of mouse synovial fibroblasts with IL-1β activated all three MAPK members, including Erk (Figure 4b, top panel), p38 (Figure 4c, top panel), and JNK1 (Figure 4d, top panel), and IL-1β significantly upregulated SYN expression (Figure 4a). These results suggest that MAPK pathways might be involved in IL-1β-induced SYN expression.

To further elucidate which MAPK pathway is involved in regulating IL-1β-induced SYN expression, specific inhibitors of MAP kinases were used [13-15,25]. Erk inhibitor, PD98059, specifically inhibited Erk phosphorylation without affecting the activation of either JNK or p38 (Figure 4b, lane 3). In the presence of this inhibitor, SYN expression was reduced to a level that is comparable with non-treated controls (Figure 4a, top panel, lane 3). p38 inhibitor, SB202190, which specifically inhibited ATF2 phosphorylation without affecting Erk and JNK1 activation, had a very mild inhibitory effect on SYN expression (Figure 4c, lane 4). The specific inhibitor of JNK, SP600125, which specifically inhibited c-Jun phosphorylation, had no effects on IL-1β-induced SYN expression (Figure 4d, lane 5). Similarly, the Erk inhibitor also inhibited TNF-α-induced SYN expression (Figure 4e). Both IL-1β and TNF-α are also strong activators of the NF-κB pathway [24]. We therefore tested whether NF-κB activation is also involved in the induction of SYN expression in mouse synovial fibroblasts. The NF-κB-specific inhibitor, SN50, strongly inhibited both IL-1β and TNF-α-induced NF-κB reporter activities (Figure 4g) but not the SYN protein expression (Figure 4f). These findings collectively indicate that IL-1β and TNF-α enhance SYN expression through the activation of Erk, but not the p38, JNK, or NF-κB pathways.

### Transcription factor ETS1 is involved in IL-1β-induced SYN expression

**Figure 5:**
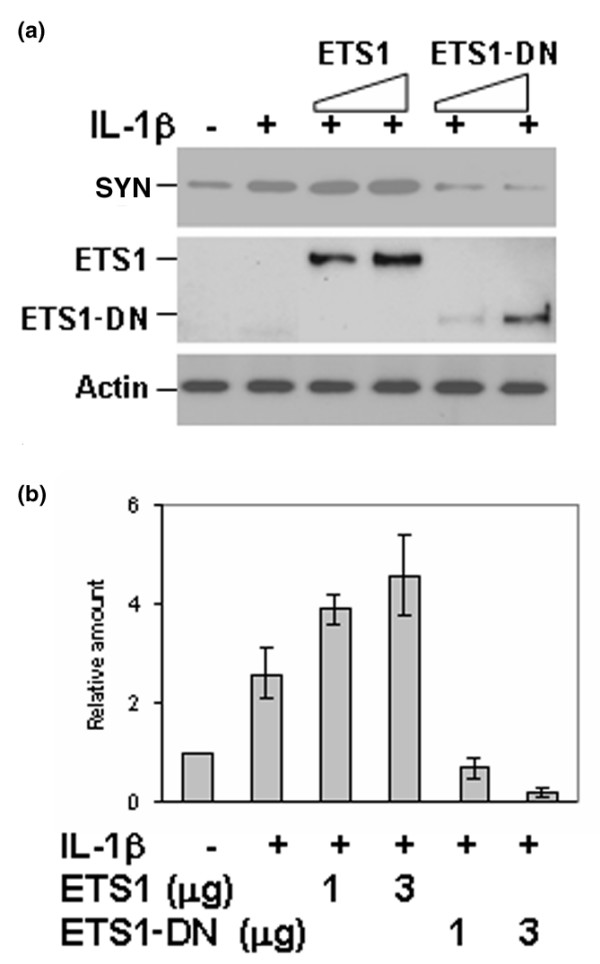
ETS1 is involved in interleukin-1β(IL-1β)-induced Synoviolin (SYN) expression. **(a) **ETS1 or ETS1-DN expression plasmids were transfected into mouse synovial fibroblasts. Cells were then cultured in medium containing 10 ng/ml of IL-1β for 48 hours. The expression of SYN was detected by Western blotting (top panel). The expression of ETS1 and its mutant was detected with anti-ETS1 antibody (middle panel). The same membrane was stripped and reblotted with anti-actin antibody (bottom panel).**(b) **The expression level of SYN was quantified. Error bars represent three independent experiments (mean ± standard deviation). ETS1-DN, dominant negative mutant of ETS1.

ETS1 is a transcription factor, the expression of which is increased in synovial fibroblasts from patients with RA [27]. Recently, the ETS binding site (EBS), termed EBS-1, from position -76 to -69 of the proximal promoter, was identified as being responsible for SYN expression [28]. Interestingly, we found that overexpression of ETS1 further enhanced IL-1β-induced SYN expression in a dose-dependent manner (Figure 5a, lanes 3 and 4; Figure 5b). To further confirm the involvement of ETS1 in SYN expression, we generated a C-terminal truncate mutation of ETS1, which has been shown to be a dominant negative mutant of ETS1 (ETS1-DN) [29]. As shown in Figure 4a (lanes 5 and 6), ETS1-DN dramatically blocked SYN expression induced by IL-1β in mouse synovial fibroblasts. These results indicate that the transcription factor is involved in regulating IL-1β-induced SYN expression in mouse synovial fibroblasts.

### Activation of Erk and ETS1 is involved in the overgrowth of synovial fibroblasts from CIA mice

**Figure 6:**
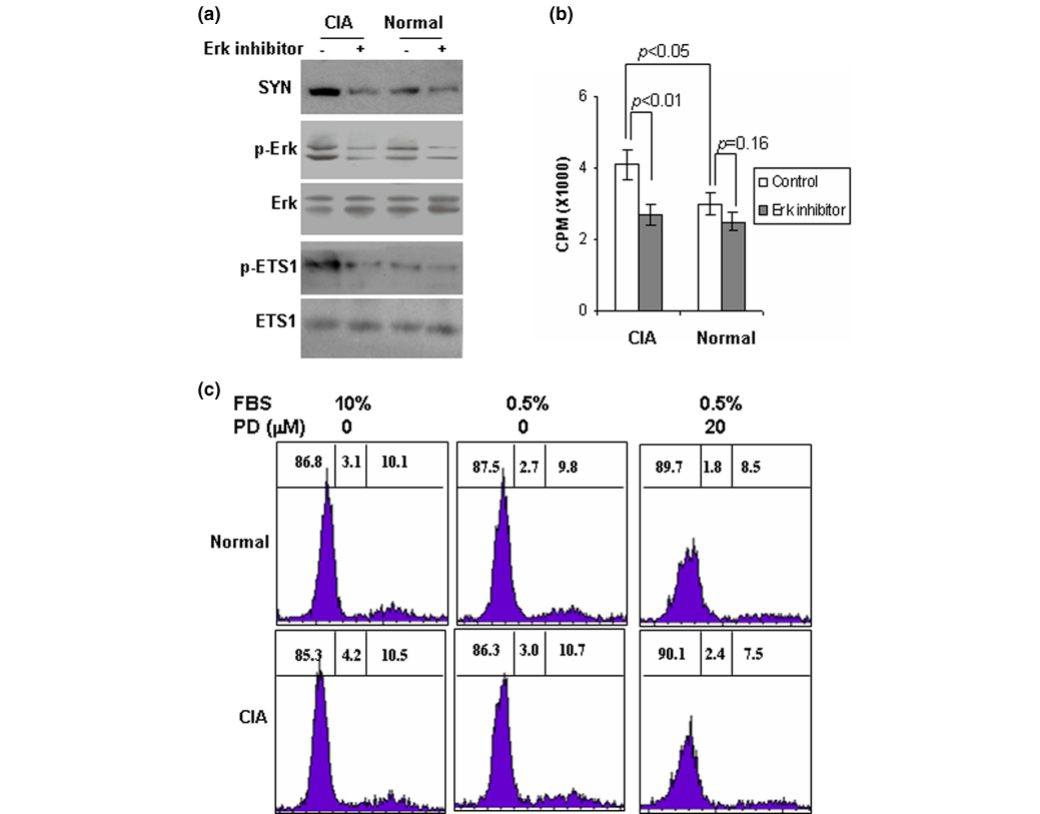
Erk inhibition suppresses ETS1 activation and hyperproliferation of mouse synovial fibroblasts from collagen-induced arthritis (CIA) mice. **(a) **Synovial fibroblasts isolated from CIA or normal mice were cultured without or with Erk inhibitor. The expression of Synoviolin (SYN) was determined by Western blotting by using anti-SYN antibody (top panel). The activation and protein levels of Erk in the parallel prepared cell lysates were detected by anti-phospho-Erk and anti-Erk, respectively (the second and third panels). Similarly, the activation and protein levels of ETS1 in the parallel prepared cell lysates were detected by anti-phospho-ETS1 and anti-ETS1, respectively (the bottom two panels). **(b) **Synovial cells were cultured without or with Erk inhibitor in 12-well plates for 24 hours. One microcurie of ^3^H-tymidine was added to each well of plated cells and further cultured for 16 hours. ^3^H-tymidine incorporation was analyzed as described previously [18]. Error bars represent three independent experiments (mean ± standard deviation). **(c) **Cell cycle analysis of Erk inhibitor-treated synovial fibroblasts. Cells treated with or without the Erk-specific inhibitor were collected and fixed in cold methanol and then stained with propidium iodide (PI) in the presence of RNase. PI-stained cells were washed once with phosphate-buffered saline and then analyzed by flow cytometry. The cell death was significantly increased when CIA synovial fibroblasts were treated with Erk inhibitor, PD98059 (PD). CPM, counts per minute; Erk, extracellular signal-regulated kinase; FBS, fetal bovine serum.

We next tested whether the activation of Erk is enhanced in the synovial fibroblasts from CIA mice, in which SYN expression is increased. As expected, both Erk1 activation and Erk2 activation are slightly increased in CIA synovial fibroblasts compared with those from normal DBA1 mice (Figure 6a). Interestingly, in treatment with the Erk-specific inhibitor, the expression of SYN was reduced to comparable levels between normal and CIA synovial fibroblasts. These results suggest that Erk activation is involved in upregulating SYN expression *in vivo *during CIA development. It has been reported that the expression of ETS1 protein is increased in the synovial fibroblasts from patients with RA [27]. We compared the activation and protein expression of ETS1 in the synovial fibroblasts from CIA mice with those from normal DBA1 mice. As shown in Figure 6a, we found that the activation of ETS1 but not its protein expression was increased in CIA synovial fibroblasts. These results suggest that the activation of both Erk and ETS1 is involved in regulating the expression of SYN in mouse synovial fibroblasts during CIA.

Based on previous studies that have suggested that Erk may activate ETS1 transcription activity [30] together with our findings that Erk and ETS1 are involved in IL-1β-induced SYN expression, we proposed that IL-1β induces SYN expression in mouse synovial fibroblasts via the Erk-ETS1 pathway. To support this hypothesis, we found that inhibition of Erk activation by Erk-specific inhibitor blocked ETS1 phosphorylation (Figure 6a). Moreover, Erk inhibitor suppressed the cell growth of mouse synovial fibroblasts, and the synovial fibroblasts from CIA mice were more sensitive to Erk inhibition (Figure 6b). Treatment of these cells with the Erk inhibitor did not significantly arrest the cell cycle of these synovial fibroblasts from either normal DBA or CIA mice (Figure 6c). These findings suggest that the activation of Erk and ETS1 is involved in the overgrowth of mouse synovial fibroblasts during CIA. Based on these findings, we collectively concluded that the Erk-ETS1 pathway is involved in SYN expression in mouse synovial fibroblasts induced by IL-1β and TNF-α.

## Discussion

Proinflammatory cytokines, particularly IL-1β and TNF-α, can induce the cell proliferation of synovial fibroblasts both *in vitro *and *in vivo *[31,32]. The molecular mechanisms of proinflammatory cytokine-induced cell growth of synovial tissues have been extensively investigated, although there still is room for debate [33]. IL-1β and TNF-α are key activators of the many transcription factors, including activator protein-1 (AP-1), Egr-1 (early growth response-1), and NF-κB, in synovial fibroblasts. Activation of the NF-κB/Rel transcription family and AP-1 complexes, composed of members of the Jun and Fos families, contributes to the hyperproliferation of fibroblast-like synoviocytes [34]. We found that IL-1β induces the proliferation of synovial fibroblasts by upregulating SYN expression. These findings provide a new mechanism for IL-1β in synovitis during RA development.

MAPKs are especially important in synovitis because they control the proliferation of synovial cells in the rheumatoid joint and induce the production of MMPs and cytokines that participate in the rheumatoid process [33,35]. The involvement of all three MAPK family members, JNK, Erk, and p38, in RA has been indicated by the fact that their activation is increased in rheumatoid synovial cells. They have also been implicated in the pathogenesis of RA [33]. The binding of IL-1β to its receptor expressed on the surface of synovial fibroblasts activates all three MAPK members, as demonstrated in our study and previous reports [33]. Intriguingly, only Erk activation is involved in SYN transcription, as indicated by the finding that the specific inhibitors against Erk, but not JNK and p38, block SYN expression. It has been demonstrated that Erk activation is highly increased in the synovial fibroblasts from patients with RA. Consistent with this, we found that the activation of Erk is significantly upregulated in mouse synovial fibroblasts from CIA mice compared with that from normal DBA1. Interestingly, the CIA synovial fibroblasts are more sensitive to Erk inhibitor-mediated cell growth inhibition. Therefore, Erk inhibitors could be potential candidates for RA treatment.

Recently, EBS-1 was identified as a crucial site for the expression of SYN [28]. EBS-1 is a binding site for ETS family transcription factors, including ETS1/2, GABP (growth-associated binding protein)-α/β, and Sp-1 [36]. Our findings that ETS1 expression increases SYN transcription and that ETS1-DN blocks SYN expression (Figure 4) confirm that the EBS-1 binding site is the promoter region for IL-1β-induced SYN expression. ETS1 is activated by Erk-mediated phosphorylation [37]. Therefore, inhibition of Erk activation by specific inhibitors blocks SYN transcription (Figure 3). Overexpression of ETS1 family transcription factors has been observed in RA synovial membranes [19,27]. However, we found that the activation of ETS1 but not its protein expression is upregulated in the synovial fibroblasts from CIA mice. The elevated ETS1 activation is possibly a direct consequence of Erk activation, because inhibition of Erk activation blocks ETS1 phosphorylation.

As an E3 ubiquitin ligase on the ER membrane, SYN functions as an ER-associated degradation system in both yeast and mammals [8,9]. The biological functions of SYN were analyzed in SYN transgenic mice and heterozygous knockout mice because the homozygous mice are embryonic-lethal [11]. Interestingly, the expression level of SYN correlates significantly with the onset of arthropathy: Increased SYN expression causes synovium overgrowth and spontaneous arthropathy, whereas reduced SYN expression (heterozygous mutant mice) is associated with resistance to CIA [11]. Our finding that the suppression of SYN inhibits IL-1β-induced synovial fibroblast proliferation provides a direct rationale for SYN as a potential target for the treatment of RA. It will be extremely interesting to investigate the effects of gene therapeutic delivery of dominant-negative SYN or its siRNA (small interfering RNA) on the development of arthritis in animals such as DBA mice with CIA.

## Conclusion

This study demonstrated that the proinflammatory cytokines IL-1β and TNF-α induce synovial fibroblast growth by upregulating the expression of an E3 ubiquitin ligase, SYN. At the molecular level, activation of MAPK Erk and transcription factor ETS1 is required for SYN expression. Based on our findings and the fact that synovial fibroblasts are among the major resources for IL-1β and TNF-α in the rheumatoid joints [31], we proposed a positive feedback model for SYN in RA development (Additional file 2). According to this model, IL-1β induces SYN transcription, SYN enhances IL-1β-induced synovial fibroblast proliferation, and the massive growth of synovial fibroblasts produces more IL-1β for the induction of SYN expression. This positive feedback process may be critical in RA development. Molecules in this signal pathway can therefore be potential targets against RA.

## Abbreviations

AP-1 = activator protein-1; CFA = complete Freund's Adjuvant; CIA = collagen-induced arthritis; DMEM = Dulbecco's modified Eagle's medium; EBS = ETS binding site; ER = endoplasmic reticulum; Erk = extracellular signal-regulated kinase; ETS1-DN = dominant negative mutant of ETS1; FBS = fetal bovine serum; GST = glutathione S-transferase; IACUC = institutional animal care and use committee; IL = interleukin; JNK = c-Jun N-terminal kinase; MAPK = mitogen-activated protein kinase; NF-κB = nuclear factor-kappa B; NP-40 = Nonidet P-40; PCR = polymerase chain reaction; RA = rheumatoid arthritis; RT-PCR = reverse transcription-polymerase chain reaction; SYN = Synoviolin; TNF-α = tumor necrosis factor-α; Ubc = ubiquitin-conjugation enzyme.

## Competing interests

The authors declare that they have no competing interests.

## Authors' contributions

BG conceived and performed most of the experiments, including anti-SYN antibody generation and signal transduction analysis. KC participated in drafting the manuscript. DF designed and organized the study, drafted the manuscript, and performed experiments in inducing arthritis and isolating synovial fibroblasts. All authors read and approved the final manuscript.

## Supplementary Material

Additional file 1 Collagen-induced arthritis in DBA/1 mice. DBA/1 mice at the age of 6 weeks were immunized with 100 μg of collagen in complete Freund's Adjuvant on day 0 and boosted with 100 μg of collagen in incomplete Freund's Adjuvant on day 21. Ten DBA/1 mice were used. Severity of joint inflammation **(a) **and incidence of arthritis **(b) **were scored. Click here for file

Additional file 2 A proposed model for interleukin-1β(IL-1β)-induced Synoviolin (SYN) expression in rheumatoid arthritis. IL-1β stimulates synovial fibroblasts and activates Erk. Activated Erk drives ETS1 activation for the transcription of SYN mRNA. The upregulated SYN increases the proliferation of synovial cells, which induces arthritis. The increased synovial fibroblasts produce more IL-1β and thereby facilitate the development of arthritis. Erk, extracellular signal-regulated kinase. Click here for file
