# Supplementary figures and images for: The proinflammatory cytokines IL-1β and TNF-α induce the expression of Synoviolin, an E3 ubiquitin ligase, in mouse synovial fibroblasts via the Erk1/2-ETS1 pathway
Source: Arthritis Res Ther. 2006 Nov 14;8(6):R172. doi: 10.1186/ar2081 (PMC1794516; doi:10.1186/ar2081)

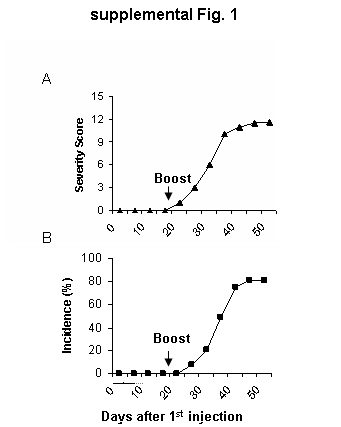

Supplement: Additional file 1 — Collagen-induced arthritis in DBA/1 mice. DBA/1 mice at the age of 6 weeks were immunized with 100 μg of collagen in complete Freund's Adjuvant on day 0 and boosted with 100 μg of collagen in incomplete Freund's Adjuvant on day 21. Ten DBA/1 mice were used. Severity of joint inflammation (a) and incidence of arthritis (b) were scored. [file ar2081-S1.tiff]

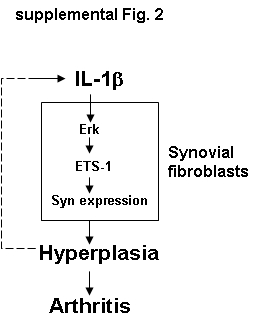

Supplement: Additional file 2 — A proposed model for interleukin-1β(IL-1β)-induced Synoviolin (SYN) expression in rheumatoid arthritis. IL-1β stimulates synovial fibroblasts and activates Erk. Activated Erk drives ETS1 activation for the transcription of SYN mRNA. The upregulated SYN increases the proliferation of synovial cells, which induces arthritis. The increased synovial fibroblasts produce more IL-1β and thereby facilitate the development of arthritis. Erk, extracellular signal-regulated kinase. [file ar2081-S2.tiff]
